# The Significance of Zinc in Patients with Chronic Liver Disease

**DOI:** 10.3390/nu14224855

**Published:** 2022-11-17

**Authors:** Hiroki Nishikawa, Akira Asai, Shinya Fukunishi

**Affiliations:** 1 Second Department of Internal Medicine, Osaka Medical and Pharmaceutical University, Takatsuki 569-8686, Osaka, Japan; 2 Premier Departmental Research of Medicine, Osaka Medical and Pharmaceutical University, Takatsuki 569-8686, Osaka, Japan

**Keywords:** zinc, chronic liver disease, zinc transporter, metallothionein, ammonia, prognosis

## Abstract

Zinc is an essential trace element for the maintenance of life because it acts as a center of activity or cofactor for hundreds of enzymes. Zinc deficiency causes a variety of symptoms, including anemia, dermatitis, stomatitis, alopecia, bedsores, decreased appetite, impaired growth, gonadal dysfunction, susceptibility to infection, and taste disorders, etc. In March 2017, zinc acetate hydrate, which had been approved for Wilson disease in Japan, received an additional indication for hypozincemia. Hypozincemia is frequently observed in patients with chronic liver disease (CLD), especially cirrhosis, and it has recently been shown that hypozincemia is closely related to the development of liver fibrosis and increased risk of liver carcinogenesis, in addition to the appearance of various subjective symptoms. Moreover, hypozincemia in CLD may be associated with sarcopenia (i.e., decrease in muscle strength and muscle mass) and frailty (i.e., vulnerability), which receive much attention these days. It is assumed that treatment with zinc acetate hydrate will become widespread in patients with CLD. Zinc acetate hydrate may also have potential for improving sarcopenia in patients with CLD. This review primarily outlines the significance of zinc in patients with CLD.

## 1. Introduction

Zinc is a trace element with atomic number 30, which is one of 16 essential minerals [1]. Zinc was shown to be essential for growth in rats in 1934 [2]. Zinc deficiency in humans was reported in 1961 [3]. The amount of zinc in the body is only about 2000 mg, and is distributed mainly in skeletal muscle, bone, skin, liver, brain, and kidney, mostly bound to proteins and other macromolecules [1,4]. Today, it is known that zinc acts as an active center or cofactor for more than 300 enzymes, and it is an essential trace element for DNA synthesis, RNA transcription, cell growth, protein synthesis, and regeneration [1,4]. In 2002, the World Health Organization (WHO) recommended that zinc deficiency is one of the most important risk factors for disease and death in developing countries. While around 17% of the world population has zinc deficiency, the frequency of zinc deficiency is around 20% in Japan, especially high among developed countries [5]. One of the reasons for this is the impact of food additives that chelate zinc, in addition to the recent aging of Japanese society, westernization of diets, and excessive dietary restrictions [6]. The percentage of Japanese who consume less than the recommended dosage of zinc increases with age [7]. Various symptoms such as dermatitis, taste disorders, neurosensory disorders, cognitive dysfunction, growth retardation, gonadal dysgenesis, immunodeficiency, chronic diarrhea, pancytopenia, stomatitis, alopecia, and anorexia are known to occur with zinc deficiency [8], and diseases causing zinc deficiency include inflammatory bowel disease, short bowel syndrome, chronic liver disease (CLD), kidney disease, and diabetes in adults [6,9]. Zinc replacement therapy has been indicated for these diseases, and in March 2017, a zinc acetate hydrate approved for Wilson disease, received an additional indication in Japan for hypozincemia [10]. While the maximum daily zinc content of zinc-containing drug for gastric ulcer is 34 mg, up to 150 mg can be administered with zinc acetate hydrate. In the treatment of hypozincemia, zinc acetate hydrate is expected to improve liver function and fibrosis, suppress liver carcinogenesis, and improve prognosis in patients with CLD. On the other hand, it has been noted that trace element deficiency in COVID-19 pandemic increases the risk of hospitalization and death [11].

This paper mainly outlines the significance of zinc in patients with CLD.

## 2. Maintenance of Zinc Homeostasis

Research on the regulatory mechanisms of zinc homeostasis has made significant progress since the late 1990s. The main absorption sites of zinc are the duodenum and jejunum, where normally 20–40% of the intake is absorbed, and zinc is maintained at about 5 mg of absorption per day, but in zinc deficiency, the absorption rate increases to around 80% of the intake [6,12]. Zinc is a trace element with a high safety margin, and hyperzincemia (serum zinc level > 130 μg/dL) is rare in daily life. Vomiting occurs at doses of 250 or more mg/day of zinc [13]. Symptoms other than vomiting, such as abdominal pain, anemia, fatigue, neutropenia, and immune dysfunction, have been reported [14], but these symptoms improve rapidly after discontinuation of zinc administration [15]. Zinc absorbed from the intestinal tract binds primarily to metallothionein. Metallothionein is a metal-binding protein rich in low molecular weight cysteine [12,16]. As zinc intake increases, metallothionein synthesis increases, zinc translocation from the extracellular to the cytoplasm occurs, the amount of zinc bound to metallothionein in the cell increases, and the efficiency of zinc absorption decreases [12]. The transfer of absorbed zinc from the intestinal tract to the bloodstream is mainly mediated by albumin [17]. Albumin is a plasma protein that has a strong ability to bind to a variety of substances, and binding substances increases the stability of albumin [17]. In addition to metallothionein, zinc transporters have been shown to play an important role in zinc homeostasis [18].

### 2.1. Zinc Transporters

Zinc is stable in vivo as a divalent cation, and its transport is mediated by zinc transporters expressed on the cell membrane. Zinc transporters, which mediate membrane permeation of zinc, were first discovered by genetic analysis in yeast, and have rapidly become better understood as a result of advances in the analysis of animal cells. Currently, there are 23 mammalian zinc transporters, which not only maintain zinc homeostasis but have been shown to have diverse physiological actions [19]. Zinc transporter is a membrane protein that transports zinc between and into cells. As a zinc transporter, it is classified into the zinc transporter gene family: (i) ZIP (Zrt-, Irt-like protein), which transports zinc from outside the cell into the cytoplasm, and (ii) Zn-T (Zn transporter), which transports zinc from inside to outside the cell [18]. Degradation and localization changes of zinc transporters occur via zinc-responsive transcription factors, which cooperate with metallothionein to maintain zinc homeostasis [18]. These zinc transporters are present in almost all organs of the body, with 9 known transporters in Zn-T and 14 in ZIP in mammalian [18]. ZIP4 plays an essential role in zinc absorption from the digestive tract (duodenum and jejunum) [18]. The consumption of foods with ZIP4 expression-promoting effects promotes the expression of ZIP4 in the intestinal tract. Attempts are being made to prevent zinc deficiency by increasing the efficiency of zinc absorption [20]. Zinc transporters also have important physiological functions related to tissue development and differentiation, and are now being shown to be closely related to the development and pathological mechanisms of various diseases [21]. ZIP4, ZIP14 and Zn-T9 have been reported to be closely related to liver carcinogenesis [21].

### 2.2. Metallothionein

Metallothionein was identified as a metal-binding protein that is induced when harmful substances such as cadmium enter the human body [16]. In addition to detoxification, metallothionein has been shown to have physiological activities such as free radical removal, inhibition of cell proliferation and differentiation, immunomodulatory functions, and metal metabolism (zinc, copper, etc.) [22,23]. Metallothionein is normally found in the cytoplasm, known as defense proteins that work to reduce metal toxicity, etc., and its localization to the nucleus occurs with cell proliferation and differentiation [24]. Zinc, cadmium, corticosteroids, endotoxin, reactive oxygen species, TNFα, IL-6, and interferon are known as metallothionein-inducing substances [25,26,27]. Metallothionein has antioxidant, cytoprotective, and hepatocyte regeneration-promoting effects on hepatocytes, but in the presence of zinc deficiency, the free radical scavenging effect of metallothionein is reduced and oxidative stress is enhanced, resulting in prolonged inflammation and inhibition of apoptosis [28]. Zinc deficiency is also thought to be associated with an increased risk of liver carcinogenesis and development of liver fibrosis [29]. In patients with cirrhosis, metallothionein expression is reduced to around 30% compared to normal liver tissue [30].

## 3. Diagnosis for Zinc Deficiency

The Japanese Society of Clinical Nutrition’s diagnostic guidelines for zinc deficiency define the following conditions as zinc deficiency: (1) One or more of the following clinical and laboratory findings are met: dermatitis, infectious disease, taste disorder, stomatitis, alopecia, bedsores, decreased appetite, growth disturbance, gonadal dysfunction, anemia, infertility, and low serum alkaline phosphatase, (2) Other diseases causing the above symptoms have been ruled out, and (3) Symptoms improve with zinc supplementation. Serum zinc levels below 60 μg/dL were defined as zinc deficiency, and serum zinc levels between 60 μg/dL and 80 μg/dL were defined as subclinical zinc deficiency [9]. In other words, a definite diagnosis is made only after symptomatic improvement with zinc supplementation. Serum zinc levels fluctuate during the day, with a high level in the early morning and a low level in the afternoon, and can increase or decrease by about 20% during the course of the day. Measurement of serum zinc level in the early morning (fasting state) can be recommended [31]. This is partly due to the excretion of zinc in the stool during the day. If serum zinc is abnormally high, hemolysis [32], ingestion of foods or drugs that promote zinc absorption (e.g., vitamin C) [33], etc. should be considered. In our study of 441 cirrhotic patients, we compared prognoses in the three groups according to baseline serum zinc levels: (a) less than 60 μg/dL (zinc deficiency group, *n* = 158), (b) 60 μg/dL to 80 μg/dL (subclinical zinc deficiency group, *n* = 227), and (c) 80 μg/dL to 130 μg/dL (normal zinc group, *n* = 56). The normal zinc group had the best prognosis among 3 groups, with a lower Akaike information criterion (the lower the value, the better the prognostic stratification) than the Child-Pugh classification [34].

## 4. Hypozincemia in Patients with CLD

In patients with CLD, zinc deficiency occurs due to decreased albumin synthesis, impaired intestinal absorption, increased urinary excretion of zinc associated with port-systemic shunt, and poor oral intake [35]. Furthermore, it has been noted that in decompensated cirrhosis, the administration of diuretics used in ascites inhibits the reabsorption of zinc from the renal tubules, contributing to zinc deficiency [35]. Serum zinc level is positively correlated with serum albumin level [34,36,37,38]. Albumin-free zinc is excreted from the urine in a hypoalbuminemic state [35,39], and the loss of zinc from the body due to hypoalbuminemia is considered the main pathogenesis of zinc deficiency resulting from advanced liver disease [35,39]. In a study of serum zinc levels in 1973 CLD cases in Japan, 89.5% of cirrhotic patients had serum zinc levels of less than 80 μg/dL and 49.8% had levels of less than 60 μg/dL [37]. On the other hand, 75.5% of non-cirrhotic patients had serum zinc levels less than 80 μg/dL, and 14.9% had serum zinc levels less than 60 μg/dL [37]. In addition, zinc deficiency (serum zinc level < 60 μg/dL) was observed in about 90% of patients with serum albumin levels < 3.5 g/dL, and in about half of the patients with serum albumin levels between 3.5 g/dL and 4.0 g/dL [37]. In our study of serum zinc levels in 472 CLD cases, 84.3% of cirrhotic patients had serum zinc levels of less than 80 μg/dL and 42.9% had serum zinc levels of less than 60 μg/dL, while 60.9% of non-cirrhotic patients had serum zinc levels of less than 80 μg/dL and 5.3% had serum zinc levels of less than 60 μg/dL [40]. It should be noted that a significant proportion of noncirrhotic patients also have hypozincemia.

### 4.1. Zinc Deficiency, Depression, Sleep Disorder and QOL in Patients with CLD

Zinc deficiency is associated with decreased QOL in patients with CLD. In our data examining the association between zinc deficiency and depression (assessed by Beck Depression Inventory-II (BDI-II) score [41]) and sleep disturbance (assessed by Japanese version of the Pittsburgh Sleep Quality Index (PSQI-J) [42]) in 322 CLD patients (121 cirrhotic cases), serum zinc levels were significantly lower in patients with higher BDI-II scores (severe depression) and in those with higher PSQI-J scores (severe sleep disturbance), which indicated the close correlation between zinc deficiency and depression or sleep disturbance [40]. In our study using the Short-Form 36 (SF-36) in patients with CLD, serum zinc levels were more closely related to the physical component summary score than to the mental component summary score [40].

### 4.2. Zinc and Viral Hepatitis

In a hepatitis C virus (HCV)-infected state, an increase in the inflammatory cytokine IL-6 promotes the translocation of zinc from the extracellular to the cytoplasm, resulting in an increase in metallothionein activity, which has antiviral activity against HCV [43]. Because direct acting antivirals (DAAs) are safe and well tolerated, they are widely administered to HCV-related cirrhotic elderly patients and have become highly effective, with a sustained virological response almost 100% [44,45]. Recently, it was reported from Japan that serum zinc increases after DAAs treatment in HCV patients [46,47]. HCV replication and proliferation activate immune cells in the liver, causing an increase in inflammatory cytokines such as IL-6 and TNFα [48]. These inflammatory cytokines suppress albumin production and increase catabolism, resulting in hypoalbuminemia [49]. DAAs suppress hepatic inflammation and inflammatory cytokines such as IL-6 [50], resulting in an increase in serum albumin and zinc levels. The increased IL-6 causes the localization of zinc to shift into the cytoplasm, resulting in a decrease in blood zinc concentration in the HCV-infected state [51]. NS3 protein, a nonstructural (NS) region of HCV, is a zinc-requiring enzyme and is involved in HCV replication [52]. NS5A protein is a zinc metallothionein and a component of replicase involved in HCV replication [53]. NS3 and NS5A proteins require zinc, but elimination of HCV renders zinc unnecessary, resulting in an increase in serum zinc level. Serum zinc level is lower in asymptomatic HCV carriers than in normal healthy individuals [35].

Although there are few reports on zinc deficiency and hepatitis B virus (HBV) infection, no difference in serum zinc concentration has been observed between HBV asymptomatic carriers and general healthy subjects [54]. To our knowledge, unlike HCV, there are no reports that indicate an important role of zinc in the proliferative process of HBV. However, Hiraoka et al. reported that serum zinc level decreases with the progression of HBV-related liver disease [55]. The effect of nucleoside analogues (NAs) on serum zinc level is unknown, but it has been shown that NA treatment increases zinc concentrations in the liver tissue in patients with HBV [56]. Zinc deficiency may be a prognostic factor in patients with HBV-related early hepatocellular carcinoma (HCC) [55].

### 4.3. Zinc Deficiency and NAFLD/NASH and Alcoholic Liver Disease

Patients with nonalcoholic fatty liver disease (NAFLD) have lower oral zinc intake than normal subjects, and nonalcoholic steatohepatitis (NASH) patients have lower oral zinc intake than NAFLD patients [57,58]. Zinc deficiency induces oxidative stress in liver mitochondria in patients with NASH, contributing to iron overload, increased insulin resistance, and liver fibrosis progression [59]. A study in NAFLD patients who underwent biopsy indicated that hypozincemia did not correlate well with the degree of inflammation activity in the liver tissue [60]. Zinc is essential for glucose metabolism homeostasis, and zinc deficiency is known to contribute to impaired glucose tolerance. Mechanisms for these include (i) zinc transporters affect insulin signaling [61], and (ii) metallothionein overexpression induced by zinc administration is thought to decrease blood glucose [62]. Metallothionein plays an important role as a susceptibility-determining gene for the amelioration of diabetes and fatty liver by zinc [61,62]. Abnormal glucose tolerance is one of the extrahepatic complications of HCV-related liver disease, and zinc deficiency was shown to induce insulin resistance in HCV patients without diabetes [63]. Ito et al. reported that serum zinc level is a predictor of the development of extrahepatic malignancies in patients with NAFLD [64].

It has long been noted that zinc deficiency is frequent in patients with alcoholic liver disease (ALD). In addition to the inhibition of zinc absorption by ethanol [65], most heavy drinkers have low zinc intake [66]. Metallothionein-transgenic mouse with intrahepatic overexpression of metallothionein and zinc was resistant to alcoholic liver injury, while metallothionein knock-out mouse with decreased intrahepatic zinc was susceptible to alcoholic liver injury [67]. Urinary excretion of zinc is increased in drinkers with or without hepatitis, and the extent of this increase further increases with the deterioration of liver fibrosis [68]. In addition to zinc, serum selenium levels are also markedly decreased in ALD [68]. The antioxidants SOD and GPX are produced in the body only in the presence of zinc and selenium [68]. On the other hand, zinc supplementation produces an increase in serum zinc and improves intestinal permeability in patients with ALD [69]. Zinc protects or strengthens the intestinal barrier function in the tight junction through multiple extracellular and intracellular mechanisms [69].

### 4.4. Zinc Deficiency and Liver Fibrosis Progression, Liver Disease-Related Adverse Events and Carcinogenesis

Zinc deficiency promotes liver fibrosis, and zinc supplementation inhibits liver fibrosis [70]. Zinc deficiency causes a decrease in the bioactivity of metallothionein and enhances oxidative stress, which has been suggested to be related to liver carcinogenesis [35]. On the other hand, there have been conflicting reports on whether zinc-deficient patients are at higher risk of liver carcinogenesis [71,72], but recently the following reports were done: (i) zinc deficiency increases liver carcinogenesis in patients with HCV-related cirrhosis, and (ii) zinc deficiency is an independent prognostic factor of liver carcinogenesis in patients with hepatitis virus-related CLD [55,73,74]. In our study of 275 HCV-related LC patients, serum zinc levels were closely associated with composite hepatic events (any one or more hepatic events such as varices, ascites, encephalopathy, carcinogenesis, etc.), with significant increase in events in the following order: 80 μg/dL ≤ baseline serum Zn level < 130 μg/dL group, 60 μg/dL ≤ baseline serum Zn level < 80 μg/dL, and baseline Zn level < 60 μg/dL [75]. A recent study demonstrated that hypozincemia (cut off value = 55 μg/dL) can be a prognostic factor of HCC incidence in patients with HCV-related cirrhotic cases [74].

### 4.5. Zinc Deficiency and Sarcopenia in Patients with CLD

Skeletal muscle mass is known to progressively decrease with age. The annual rate of decrease in skeletal muscle mass is about 1% per year for people over 50 years of age, and the annual rate of decrease is even higher for elderly people (e.g., bedridden status) whose activity level in daily life is declining. Approximately 60% of the zinc in vivo is contained in skeletal muscle, and it has recently been shown that quantitative and qualitative decreases in skeletal muscle (i.e., sarcopenia) are associated with decreased metallothionein expression [76]. In cirrhotic patients, the rate of annual loss of muscle mass has been shown to be approximately twice as high as that of the average elderly Japanese [77]. In 2016, the Japanese Society of Hepatology published criteria for secondary sarcopenia specific to liver disease [12], and several years later, a revision by the working group was considered. A prognostic study of 1624 patients with CLD showed that grip strength (GS) was an independent prognostic factor, and muscle mass was not a significant factor in the multivariate analysis. Based on these results, the reference value for GS in men was revised from 26 kg to 28 kg (women remained unchanged at 18 kg) [78]. These reference values of 28 kg for male GS and 18 kg for female GS are consistent with those proposed by the Asian Working Group for Sarcopenia (AWGS) [79].

The results of a meta-analysis on sarcopenia in cirrhotic patients were presented [80]. This meta-analysis showed that (i) the complication rate of sarcopenia in overall cirrhotic cases was 37.5%, (ii) the frequency of sarcopenia increased with worsening Child-Pugh score, (iii) the complication rate on sarcopenia was higher in alcoholic cirrhotic patients (49.6%), (iv) the 5-year survival rate was 45.3% in sarcopenic cases and 74.2% in non-sarcopenic cases, and (v) in forest plots, the upper limit of the 95% confidence interval for the hazard ratio of sarcopenia is below 1.0 in all articles, indicating that sarcopenia is indeed a prognostic factor in cirrhotic patients [80].

**Figure 1:**
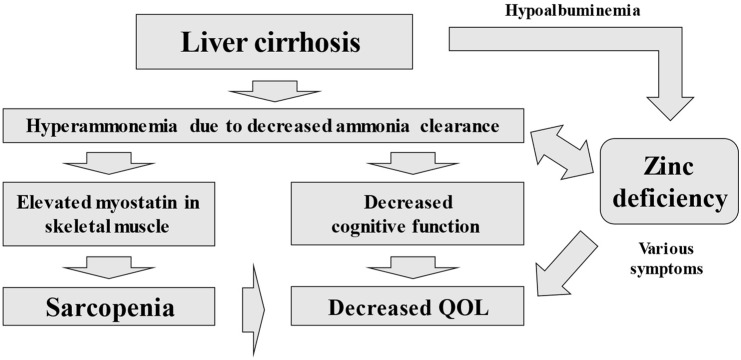
Hyperammonemia, zinc and decreased QOL in cirrhosis.

**Figure 2:**
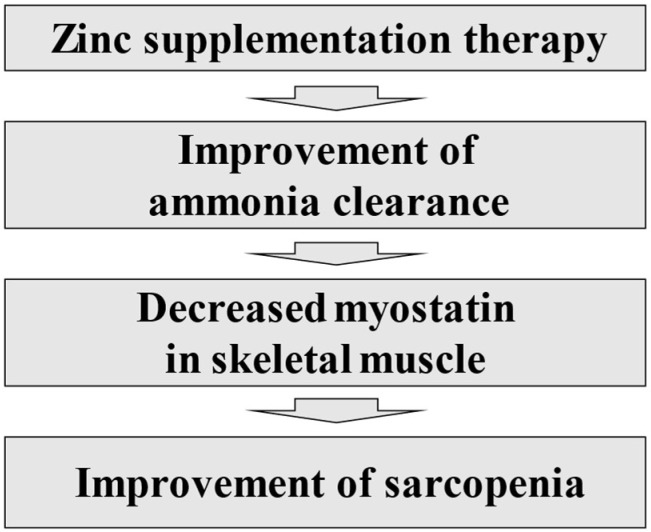
Zinc supplementation therapy and improvement of sarcopenia.

With regard to the treatment of cirrhotic cases complicated by sarcopenia, the Japanese Practice Guidelines for Cirrhosis in 2020 suggest exercise and nutritional therapy [81]. With regard to drug intervention, animal studies have suggested that improvement of hyperammonemia may lead to improvement of sarcopenia [82]. A clinical study has reported that L-carnitine improves sarcopenia via improvement of hyperammonemia in cirrhotic patients [83]. Myostatin, a myokine with inhibitory effects on skeletal muscle protein synthesis, has also been reported to be a prognostic factor in cirrhotic patients [84]. Serum myostatin and ammonia levels are positively correlated [84]. Hyperammonemia caused by decreased liver function can be associated with decreased cognitive function and decreased QOL [36]. On the other hand, the ameliorative effect of zinc supplementation on hepatic encephalopathy has been demonstrated in a meta-analysis [85]. We reported that low serum zinc level is a risk factor for sarcopenia in patients with CLDs [38]. It has also been reported that skeletal muscle index (SMI) significantly correlates with serum zinc level in cirrhotic patients [86]. The relationship between hyperammonemia, zinc, and decreased QOL in cirrhotic patients is shown in Figure 1. The authors’ concept of the association between cirrhosis complicated with sarcopenia and zinc supplementation is shown in Figure 2. Zinc supplementation in cirrhotic patients with sarcopenia is expected to improve sarcopenia, possibly by lowering myostatin in skeletal muscle from improved ammonia clearance. A recent randomized controlled trial demonstrated that branched-chain amino acid supplementation contributes to the improvement of sarcopenia and frailty partly through an increase in serum zinc level in cirrhotic patients with sarcopenia [87].

### 4.6. Zinc Deficiency and Frailty in Patients with CLD

Frailty is a state of increased vulnerability due to various age-related changes in physical functions and loss of reserve capacity [88]. It is known that frailty patients are more likely to have impaired daily functioning, falls, hospitalization, and other health problems, and have a higher mortality rate. Frailty is an important concept for estimating life and functional prognosis in the elderly and for comprehensive geriatric care [88]. Many factors are known to contribute to frailty, including sarcopenia, life-style dysfunction, immune abnormalities, and neuroendocrine abnormalities, which are all involved in a complex manner. Sarcopenia is a major component of physical frailty [88]. In our country, the frequency of frailty among community-dwelling elderly was 11.3% (mean age = 71 years) [88,89]. As is evident from the fact that CLD patients are getting older, a certain number of CLD patients should be included in the patients eligible for frailty health checkups, and frailty in CLD is a problem that cannot be overlooked in the same way as sarcopenia. Fried et al. defined frailty as meeting three or more of the following criteria: weight loss, fatigue, decreased activity, decreased mobility, and muscle weakness, and prefrail as meeting one or two [89]. Prognosis can be stratified among three groups: frailty, prefrail, and robust. In our study in patients with CLD using Fried criteria (341 patients (122 cirrhotic subjects), median age = 66 years), 46 patients (13.5%) were frailty, the frequency of frailty increased with age (*p* = 0.0002), and the frequency of cirrhotic patients in frailty patients was higher than that in non-frailty patients (67.4% vs. 30.9%, *p* < 0.0001) [90]. These results indicate that frailty in CLD, like sarcopenia, also involves a disease-specific aspect. On the other hand, with regard to the correlation between serum zinc level and frailty in CLD, in our study of 285 CLD cases (107 cirrhotic patients), serum zinc levels decreased significantly as the disease progressed from robust, prefrail, and frailty [91]. In addition, there were significant differences in serum zinc level in all of five items by Fried et al. (i.e., weight loss, fatigue, decreased activity, decreased movement speed, and decreased muscle strength), when comparing the “yes” group and “no” group [91]. These findings suggest that in patients with CLD, zinc levels are closely correlated with frailty as well as sarcopenia.

## 5. Effect of Zinc Supplementation Therapy

Ammonia is produced systemically and metabolized in the liver and skeletal muscle. Ornithine transcarbamylase (OTC), a zinc enzyme and urea circuit-related enzyme, and glutamine synthetase, a zinc enzyme, are involved in ammonia metabolism in the liver and skeletal muscle, respectively [35]. When OTC activity localized to mitochondria in hepatocytes is reduced by zinc deficiency, urea cycle function is also reduced, resulting in the appearance of hyperammonemia [35]. Furthermore, ammonia that cannot be processed by the urea cycle is metabolized by the glutamine synthesis system in skeletal muscle, which is also less active during zinc deficiency. Zinc replacement therapy to improve liver function to process ammonia is a logical treatment, and the usefulness of zinc administration in patients with hyperammonemia has been reported [92]. Serum zinc and ammonia levels are inversely correlated (correlation coefficient *r* = −0.34424, *p* < 0.0001, *n* = 426, in our data). Zinc acetate hydrate is effective in hyperammonemic patients with hepatic encephalopathy [93].

In a study of patients with hepatic encephalopathy, the number connection test (NCT) and digit symbol test (DSA) were used to evaluate the effect of zinc replacement therapy on quality of life, and the zinc replacement therapy group showed significant improvement [94]. A sub-analysis showed that the number of patients with ascites decreased, albeit at a tendency (*p* > 0.05) [94]. In a meta-analysis of the effects of zinc supplementation on hypoalbuminemia associated with cirrhosis, improvement in hypoalbuminemia was not shown [85]. Although there are few reports examining zinc replacement therapy and survival, a study analyzing the impact of zinc replacement therapy on the incidence of HCC found that events such as death, development of HCC, and liver failure were significantly lower in the zinc replacement therapy group [95]. In a study on the survival of zinc acetate hydrate in patients with decompensated liver cirrhosis with hypozincemia, patients with improved serum zinc levels were reported to have a better prognosis [96]. On the other hand, serum zinc levels have been shown to improve dose-dependently with zinc replacement therapy to serum zinc levels of 80 μg/dL or higher in many cases after 6 months of treatment while maintaining drug adherence [10,93]. Although few serious adverse events have been observed with zinc acetate hydrate, hypocopperemia has been observed in a certain percentage of patients. In particular, patients with a Child-Pugh score of 8 or higher prior to zinc acetate hydrate therapy have a high incidence of hypocopperemia and require caution [93]. Additionally, zinc acetate hydrate is more likely to cause hypocopperemia than polaprezinc [97]. When administering zinc acetate hydrate for long periods of time, it is important to regularly monitor serum copper level as well as serum zinc level, keeping in mind the possibility of hypocopperemia.

## 6. Final Remarks

The relationship between zinc and CLD is outlined based on the latest findings. Various evidences of zinc on liver fibrosis, liver carcinogenesis, sarcopenia, etc. have been accumulated, bringing great gospel to clinical practice. Further evidence is expected in the future.

## Abbreviations

CLD	chronic liver disease
ZIP	Zrt-, Irt-like protein
Zn-T	Zn transporter
BDI-II	Beck Depression Inventory-II
PSQI-J	Japanese version of the Pittsburgh Sleep Quality Index
HCV	hepatitis C virus
DAA	direct acting antiviral
NS	nonstructural
HBV	hepatitis B virus
NA	nucleoside analogue
NAFLD	nonalcoholic fatty liver disease
NASH	nonalcoholic steatohepatitis
ALD	alcoholic liver disease
GS	grip strength
OTC	Ornithine transcarbamylase
HCC	hepatocellular carcinoma
